# Abnormal Regional Homogeneity in Patients With Obsessive-Compulsive Disorder and Their Unaffected Siblings: A Resting-State fMRI Study

**DOI:** 10.3389/fpsyt.2019.00452

**Published:** 2019-06-28

**Authors:** Xiangyun Yang, Jia Luo, Zhaoxi Zhong, Xiaojie Yang, Shumin Yao, Pengchong Wang, Jian Gao, Rui Liu, Jing Sun, Zhanjiang Li

**Affiliations:** ^1^The National Clinical Research Center for Mental Disorders & Beijing Key Laboratory of Mental Disorders, Beijing Anding Hospital, Capital Medical University, Beijing, China; ^2^Advanced Innovation Center for Human Brain Protection, Capital Medical University, Beijing, China; ^3^Henan Key Lab of Biological Psychiatry, the Second Affiliated Hospital of Xinxiang Medical University, Xinxiang, China; ^4^School of Medicine, Griffith University, Gold Coast, QLD, Australia

**Keywords:** obsessive-compulsive disorder, sibling, regional homogeneity, resting-state functional magnetic resonance imaging, neuroimaging endophenotype

## Abstract

**Objective:** Previous studies suggest that abnormal brain structure and function may be neuroimaging endophenotypes of obsessive-compulsive disorder (OCD). Comparing the intrinsic brain activity of OCD patients and their unaffected siblings will help to further understand the susceptibility to, and pathological mechanisms of, OCD. We used a case–control study design aiming to establish whether the abnormal regional homogeneity (ReHo) found in OCD patients also exists in their unaffected siblings.

**Method:** Fifteen unmedicated OCD patients, 15 of their unaffected siblings, and 30 healthy controls (HCs) received resting-state functional magnetic resonance imaging (r-s fMRI) scanning and clinical evaluation. We used the ReHo method to analyze the inter-regional synchronized activity of all participants. One-way analysis of covariance with post hoc tests was used to compare the ReHo maps across groups. A Pearson correlation analysis was conducted to assess the correlations between clinical characteristics and abnormal ReHo in OCD patients.

**Results:** Relative to HCs, OCD patients and their unaffected siblings showed overlapping higher ReHo values in the right dorsolateral prefrontal cortex (DLPFC). Patients with OCD showed increased ReHo in left middle frontal gyrus (MFG) relative to both their unaffected siblings and HCs. In addition to the right DLPFC and left MFG, OCD patients, compared with HCs, also showed abnormal ReHo in other regions, including higher ReHo in the right superior parietal cortex and lower ReHo in the left inferior parietal cortex, right parahippocampal region, left thalamus, and right inferior temporal cortex. Compared with HCs, the unaffected siblings of patients with OCD had significantly higher ReHo in the right inferior parietal cortex, right MFG, and right supplementary motor area. There was no association between clinical symptoms and abnormal ReHo values in OCD patients.

**Conclusions:** This study found overlapping higher ReHo values in the right DLPFC of OCD patients and their unaffected siblings. Our results suggest that the higher ReHo in the right DLPFC may be a potential neuroimaging endophenotype, which may reflect an increased genetic risk of OCD.

## Introduction

Obsessive-compulsive disorder (OCD) is the fourth most common mental illness affecting individuals from childhood through adult life, and it creates a heavy economic burden for patients and their families (1). People with OCD may experience a chronic course of illness, which is protracted and seriously impairs their social, professional, and learning functions (2). Despite the high morbidity associated with OCD, the pathophysiology of the disorder remains unclear.

Investigations into the etiology of OCD suggest that the disorder has a genetic basis. The prevalence of OCD among the first-degree relatives of children and adolescents with OCD is reported as being about 7–15% (3). Twin studies have shown that OCD is heritable, and childhood onset has a greater genetic impact than has adult onset of OCD (45–65% vs. 27–47%) (4). One review on imaging and the genetics of OCD suggests that intrinsic brain activity may be under genetic control (5). Therefore, exploring neuroimaging phenotypes may bridge the gap in our knowledge between genetic predisposition and clinical symptoms. Unaffected siblings, who have similar family endophenotypes, provide a better opportunity to explore the endophenotype of OCD. Hence, integrating neuroimaging and genetic methods to analyze the phenotypes of OCD patients and the endophenotype of unaffected relatives is an effective way to study the pathological mechanism of OCD.

Magnetic resonance imaging (MRI) has been widely used to detect neuroimaging endophenotypes of patients with OCD and their relatives. Structural MRI studies have demonstrated that patients with OCD and their first-degree relatives exhibited gray matter and white matter abnormalities in common regions, including the orbitofrontal cortex, medial frontal region, cingulum bundle, and striatum, and these abnormalities mainly exist in the orbito-frontal-striato-thalamic and posterior brain circuits (6, 7). These findings suggest that there may be structural endophenotypes that represent genetic markers of the increased risk for OCD (8, 9). Furthermore, a study combined a response inhibition task (stop signal) and structural MRI to detect the neurocognitive endophenotypes in OCD patients and their unaffected first-degree relatives. This study indicated that changed gray matter density in brain regions including the orbitofrontal, right inferior frontal, cingulate, parietal, and subcortical regions were related to motor inhibitory control, providing evidence of neurocognitive endophenotypes of OCD (10).

Several task-related functional MRI (fMRI) studies have detected brain functional changes in OCD patients and their relatives. One study performed fMRI scanning during an emotion regulation task in OCD patients and their unaffected siblings to test whether emotion regulation was involved in genetic risk for OCD. However, they did not find the same degree of distress and amygdala activation during emotional provocation in OCD patients and their siblings (11). Chamberlain and colleagues found abnormally reduced activation in the orbitofrontal cortex, lateral prefrontal cortex, and parietal during reversal learning in both patients with OCD and their healthy first-degree relatives (12). Another study using a stop-signal task suggests that presupplementary motor area hyperactivity is a neurocognitive endophenotype of OCD (13). de Vries and colleagues performed a visuospatial *n*-back task fMRI to compare the brain activity in OCD patients and their siblings with HCs, and they found that frontoparietal brain activity may be an endophenotype of OCD (14). Matilde et al. found decreased activity of cortical regions and dysconnectivity of frontostriatal regions during an executive task (goal-directed planning) in both OCD patients and their first-degree relatives (15). The current limited results suggest that specific regions showing abnormal function during cognitive tasks may be neuroimaging endophenotypes of OCD. However, these task-related studies only focused on specific brain regions and may have missed important information existing in regions not related to the task.

Resting-state fMRI (r-s fMRI) can explore the intrinsic spontaneous brain activity of the whole brain without any hypothesis being advanced (16). Until now, only two studies have used r-s fMRI to detect the neuroimaging endophenotypes of OCD. One r-s fMRI study performed in China found that both patients with OCD and their relatives exhibited higher functional connectivity in the bilateral caudate nucleus, left orbitofrontal cortex, and left middle temporal gyrus (17). de Vries and colleagues also investigated the functional connectivity within the frontoparietal network, cingulo-opercular network, and fronto-limbic network in the resting state in unmedicated OCD patients, unaffected siblings, and HCs. They only found increased functional connectivity within and between networks in siblings, which may indicate that enhanced cognitive control may be a protective factor for OCD (18). The two preliminary studies that focused on functional connectivity show that resting-state brain function has an important value in exploring neuroimaging endophenotypes of OCD.

Regional homogeneity (ReHo), as a reliable method to measure the local connectivity of a given voxel and its neighboring voxels, has been used to explore the coordination of spontaneous brain activity in neuropsychiatric disorders, including OCD (19, 20). Compared with other methods for analyzing spontaneous brain activity, such as seed-region-based functional connectivity and amplitude of low-frequency fluctuation (ALFF), ReHo is more stable during test–retest analysis and is less affected by global nuisances (21). Some studies have used ReHo to detect the brain function of OCD patients and found important regions related to OCD, including the prefrontal (including the anterior cingulate gyrus), temporal, parietal, occipital lobes, basal ganglia, cerebellum and limbic system (22–24). In addition, a recent study used machine learning to explore the value of four r-s fMRI metrics including ALFF, fractional ALFF, ReHo, and functional connectivity in differentiating patients with OCD from normal controls. The study findings have shown that ReHo has good performance in distinguishing OCD patients from normal controls (25).

Based on the above advantages, ReHo may be a better metrics for exploring the neuroimaging endophenotypes of OCD. As a data-driven method without any prior hypothesis, ReHo can be used to obtain more information throughout the whole brain (20). To our knowledge, to date, no study has assessed ReHo differences between OCD patients and their siblings. The current study aims to identify neuroimaging endophenotypes by examining whole-brain ReHo in the resting state in patients diagnosed with OCD, their unaffected siblings, and age- and education-matched HCs.

## Methods

### Participants

A case–control study design involving 15 patients diagnosed with OCD, 15 unaffected siblings of these patients, and 30 HCs was used for the study. OCD patients were recruited from Beijing Anding Hospital in China from January 2013 to October 2016. All patients were diagnosed as OCD according to the criteria of *Diagnostic and Statistical Manual of Mental Disorders, Fourth Edition* (DSM-IV) by psychiatrists using the Structured Clinical Interview for DSM-IV Axis I Disorders (SCID). The right-handed subjects aged between 18 and 45 years were included. Yale–Brown Obsessive-Compulsive Scale (Y-BOCS) (26), 14-item Hamilton Rating Scale for Anxiety (HAM-A), and 17-item Hamilton Depression Rating Scale (HAMD-17) were used for assessing the clinical symptoms (27). Only OCD patients with Y-BOCS score of 16 or more and HAMD score of less than 18 can be included. Patients with history of neurological illness or other major physical illness, Axis I psychiatric disorders other than OCD, psychoactive substance use, alcohol dependence, and/or alcohol abuse and contraindications to MRI were excluded. Female patients who were pregnant or had intention to become pregnant were also excluded. Since psychotherapy or physical therapy may affect brain functions, patients who had ever accepted a relevant treatment were also excluded. Of the 15 participants with OCD, 12 were drug naïve and 3 had a history of treatment with selective serotonin reuptake inhibitors but had stopped their medication for more than a month prior to entering the study.

Unaffected siblings of patients with OCD were recruited by approaching patients in the clinic. Thirty healthy participants were recruited from the local community as HCs. To increase the statistical power, a ratio of two HCs to one OCD patient was used to recruit the HCs. Both HCs and siblings of patients with OCD were aged between 18 and 45 years, were right handed, and have matched education with patients with OCD. They also received diagnostic interviews with psychiatrists using the Structured Clinical Interview for DSM-IV Axis I Disorders—Non-patient Edition. Exclusion criteria included 1) a history of psychiatric or neurologic illnesses; 2) psychoactive substance use; 3) alcohol dependence and/or alcohol abuse; 4) serious physical illness; 5) being pregnant or have plans to become pregnant; and 6) contraindications to MRI.

Current study protocol was approved by the Research Ethics Committee of Beijing Anding Hospital, Capital Medical University, China. All participants provided written informed consent, in compliance with regulations of the institution and the guidelines of the Declaration of Helsinki.

### Measurements and Evaluation

We assessed the severity of OCD symptoms using the Chinese edition of the Y-BOCS. Current symptoms of depression and anxiety were assessed using the HAMD-17 and the 14-item HAM-A (28), respectively. Two psychiatrists who received training independently assessed the participants with the above instruments.

### Data Acquisition

#### MRI Scanning and ReHo Analysis

We performed the MRI scanning at the State Key Laboratory of Cognitive Neuroscience and Learning, Beijing Normal University, China. A Siemens Trio 3-Tesla scanner (Siemens Magnetom Trio; Erlangen, Germany) was used for all scans. The scanning sessions included localization, 3D T1 anatomical, conventional T2 imaging, and r-s fMRI sequences. To maintain a resting state, subjects were instructed to remain relaxed during the scanning process, keep their head still, close their eyes but not to fall asleep, and not to think about special things (16). The images of r-s fMRI scans were obtained using an echo-planar imaging sequence (the period was 8 min). The parameters included repetition time (2,000 ms), echo time (30 ms), flip angle (90°), thickness/gap (3.5/0.6 mm), field of view (200 × 200 mm), matrix (64 × 64), and 240 volumes. All images were examined by an experienced radiologist after scanning.

Image preprocessing and ReHo analysis were carried out using Data Processing and Analysis for (Resting State) Brain Imaging (DPABI) (29). The preprocessing included removing the first 10 time points, slice timing correction, head motion correction, nuisance covariate regression (including the white matter signal, cerebrospinal fluid signal, and Friston 24-parameter model), normalization, removing linear trends, and filter. After these preprocessing steps, r-s fMRI data were used for ReHo calculation. Finally, a spatial smoothing was performed for the standardized ReHo maps using a Gaussian kernel of 4 mm. Details of the whole analysis of ReHo have been described in our previous studies (30).

#### Statistical Analysis

To detect differences in the demographic and clinical data, we compared the age, gender, years of education, Y-BOCS score, HAMD-17 score, and HAM-A score among the three groups by conducting one-way analysis of variance (ANOVA) and the χ^2^ test using SPSS software version 23.0. The statistical significance was set at *P* value less than 0.05.

For the imaging data, statistical analyses were performed according to the guidelines of the statistical module of DPABI-V 2.3 (29). A one-way analysis of covariance (ANCOVA) was conducted to detect the group differences in the ReHo maps across the three groups with age, gender, and years of education as covariates. The corrected *P* value for ANCOVA was set at *P* < 0.05 using the Gaussian random field (GRF) method (voxel *P* value < 0.001, cluster *P* value < 0.05). Particularly, DPABI also offers a *post hoc* comparison correction over group pairs after ANCOVA. We used Bonferroni correction for this *post hoc* procedure. The *P* maps were then converted to *Z* maps with the sign of group mean differences. Using the *Z* maps, we then performed GRF correction to correct multiple comparisons over voxels (voxel *P* value < 0.001, cluster *P* value < 0.05). In order to show the group differences, we then extracted the mean ReHo value of all regions showing significant group differences to draw scatterplots. Finally, to determine whether abnormal ReHo is associated with clinical characteristics (a measured by the Y-BOCS score, HAMD-17 score, and HAM-A score) of OCD, we conducted a voxel-based correlation analysis in OCD patients, compared with HCs, within regions showing significant ReHo differences, when age, gender, and years of education as confounding factors were controlled. A threshold of *P* = 0.05 was used to determine the significance level.

## Results

### Comparison of Demographic and Clinical Data Among Three Groups

As shown in **Table 1**, the three groups (15 participants with OCD, 15 unaffected siblings, and 30 HCs) were not statistically different for age (*F* = 0.02, *P* = 0.98), gender (χ^2^ = 1.96, *P* = 0.38), and years of education (*F* = 2.13, *P* = 0.13). *Post hoc* tests did not find significant differences between groups. The illness duration of participants with OCD was 85.77 ± 46.9 months. There were significant differences in clinical measures (Y-BOCS total scores, obsessions scores, compulsions scores, HAMD scores, and HAM-A scores) among the three groups. *Post hoc* tests found that OCD patients had higher scores in total for the Y-BOCS, obsessions, compulsions, HAMD, and HAM-A than had the HCs or the siblings (all *P* < 0.001). There were no significant differences in clinical measures between siblings and HCs (all *P* > 0.05).

**Table 1:** Demographic and clinical characteristics of OCD patients, unaffected siblings, and healthy controls (HCs).

Variables (M ± SD)	OCD patients (*n* = 15)	Unaffected siblings (*n* = 15)	Healthy controls (*n* = 30)	*F*/χ^2^	*p*
Age (years)	28.77 (6.84)	28.38 (8.38)	28.23 (7.78)	0.02	0.98^#^
Gender (female:male)	9:6	6:9	10:20	1.96	0.38^#^
Education (years)	12.46 (3.92)	12.15 (3.78)	13.47 (2.99)	2.13	0.13^#^
Illness duration (months)	85.77 (46.9)	—	—	—	—
Y-BOCS score					
Total	25 (6.29)	0.77 (1.01)	0.27 (0.58)	322.27	<0.001*
Obsessions	12.15 (5.49)	0.38 (0.65)	0.17 (0.37)	101.38	<0.001*
Compulsions	12.92 (3.54)	0.39 (0.62)	0.10 (0.40)	267.17	<0.001*
HAMD-17 score	7.23 (4.74)	0.27 (1.54)	0.3 (0.95)	37.68	<0.001*
HAM-A score	6.54 (4.21)	1.62 (2.36)	1.57 (0.73)	23.57	<0.001*

Data are expressed as mean and SD. SD, standard deviation; OCD, obsessive-compulsive disorder; Y-BOCS, Yale–Brown Obsessive-Compulsive Scale; HAMD: the 17-item Hamilton Depression Rating Scale; HAMA, Hamilton Anxiety Rating Scale.

^#^There was no difference for post hoc tests between any two groups.

*For all post hoc tests, OCD patients differed significantly from siblings and controls, but siblings and controls were not significantly different.

### ReHo Differences Among the Three Groups

**Figure 1:**
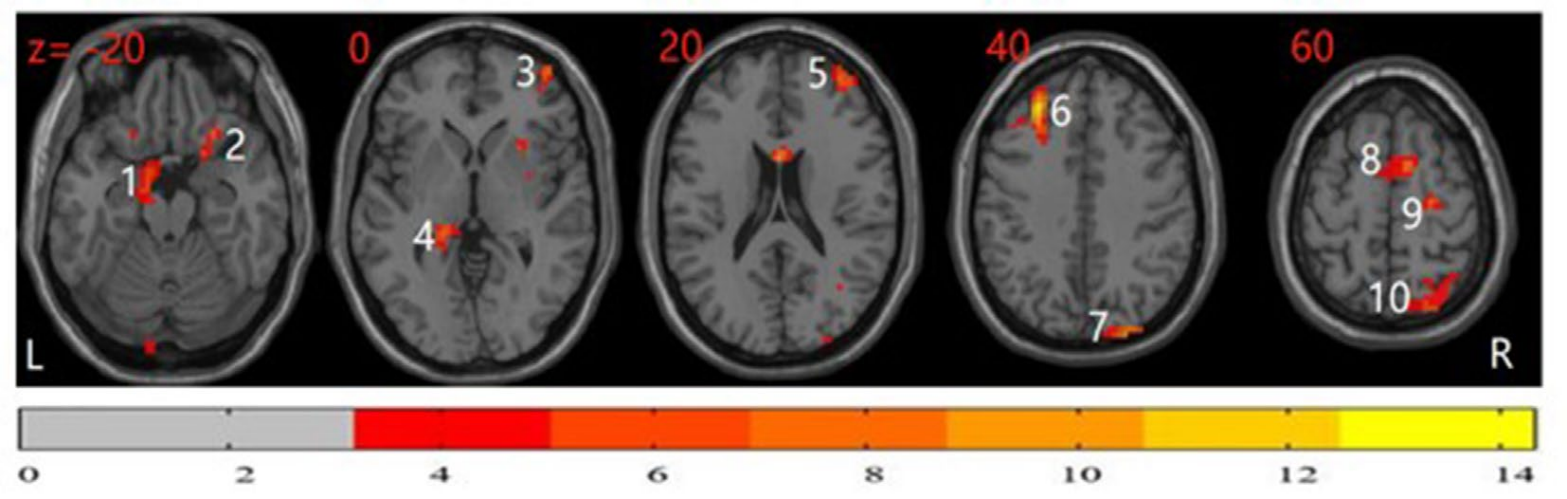
Analysis of covariance (ANCOVA) map of regional homogeneity (ReHo) among patients with obsessive-compulsive disorder (OCD), unaffected siblings of OCD patient, and healthy controls (HCs) (*P* < 0.05). The significant differences among the three groups primarily exist in the bilateral frontal cortex, cingulate cortex, parietal area, occipital cortex, and temporal cortex. The image shows the transverse plane of the brain. The color bar shown on the bottom represents *F* score. 1: left parahippocampal; 2: right inferior orbitofrontal cortex; 3: right middle frontal cortex; 4: left hippocampus; 5: right middle frontal cortex; 6: left middle frontal cortex; 7: right superior occipital cortex; 8: right supplementary motor area; 9: right superior frontal cortex; 10: right superior parietal cortex.

The differences in ReHo among the three groups were compared using ANCOVA with *post hoc* analysis. As shown in **Figure 1**, significant group differences in ReHo values primarily exist in the bilateral frontal cortex, cingulate cortex, parietal area, occipital cortex, and temporal cortex (*P* < 0.05, corrected). The detailed information for all regions with abnormal ReHo in the three groups is shown in **Table 2**.

**Table 2:** Regions with significant differences in regional homogeneity (ReHo) among OCD patients, unaffected siblings, and HCs.

Regions	Side	BA	MNI coordination			Voxels	*t* value
			X	Y	Z		
OCD > HCs							
DLPFC	R	46	42	57	1	38	−3.045
MFG	L	9	−27	36	42	121	−4.45
Super parietal cortex	R	7	21	−60	69	80	−3.656
OCD < HCs							
Inferior parietal cortex	L	40	−39	−39	54	33	2.735
Inferior temporal cortex	R	20	54	3	−36	35	3.441
Parahippocampal gyrus	R	28	24	3	−30	40	2.779
Thalamus	L	—	−12	−18	8	30	2.821
OCD > siblings							
Parahippocampal gyrus	L	28	−12	0	−24	93	3.37
MFG	L	9	−27	30	36	63	3.54
Siblings > HCs							
Inferior parietal cortex	R	40	57	−45	48	83	3.823
DLPFC	R	46	39	51	18	58	3.636
Supplementary motor area	R	6	9	6	60	59	3.225

MNI, Montreal Neurological Institute; BA, Brodmann area; L, left; R, right; OCD, obsessive-compulsive disorder; HCs, healthy controls; DLPFC, dorsolateral prefrontal cortex; MFG, middle frontal gyrus.

P < 0.05.

**Figure 2:**
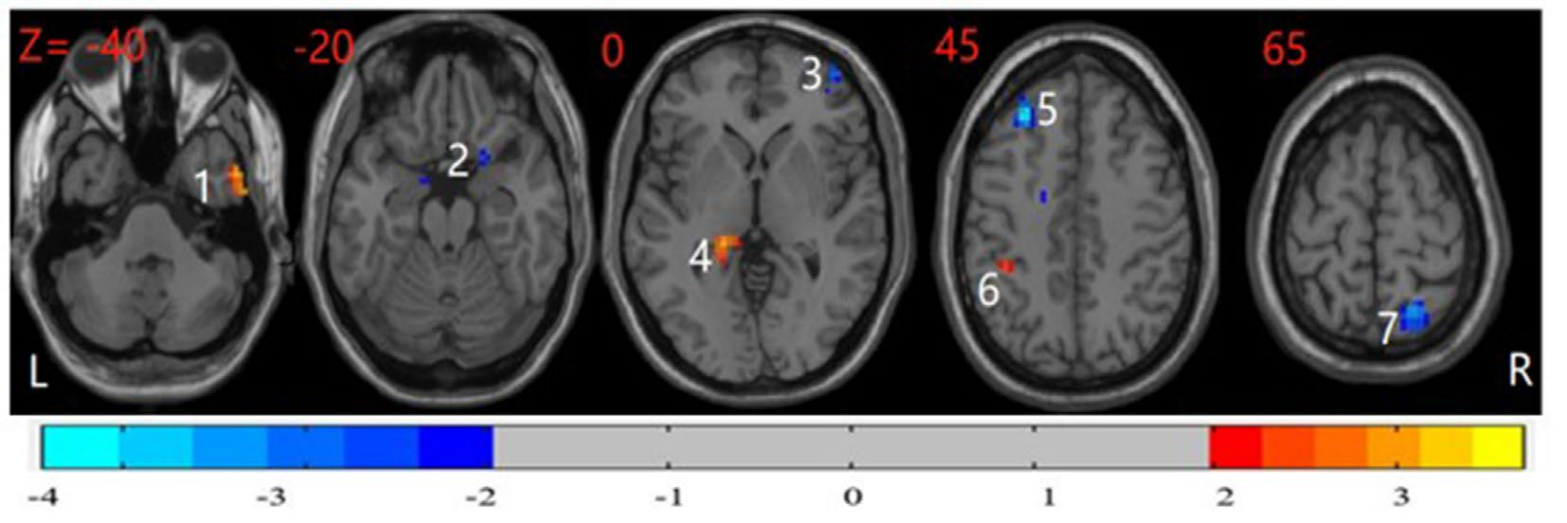
*Post hoc* test maps of ReHo between healthy controls and patients with OCD (*P* < 0.05). The healthy controls showed significantly lower ReHo in the bilateral middle frontal gyrus and right superior parietal cortex and higher ReHo in the right parahippocampal, left inferior parietal cortex, and right inferior temporal cortex. The image shows the transverse plane of the brain. Red and blue denote higher and lower ReHo comparing healthy controls with patients, respectively. L: left; R: right; 1: right inferior temporal cortex; 2: right parahippocampal gyrus; 3: right dorsolateral prefrontal cortex; 4: left thalamus; 5: left middle frontal gyrus; 6: inferior parietal cortex; 7: right superior parietal cortex.

In *post hoc* tests, compared with the HCs, participants with OCD showed significantly higher ReHo in the right dorsolateral prefrontal cortex (DLPFC), left middle frontal gyrus (MFG), and right superior parietal cortex and lower ReHo in the left inferior parietal cortex, right parahippocampal gyrus (PHG), left thalamus, and right inferior temporal cortex (*P* < 0.05, corrected) (**Figure 2**).

**Figure 3:**
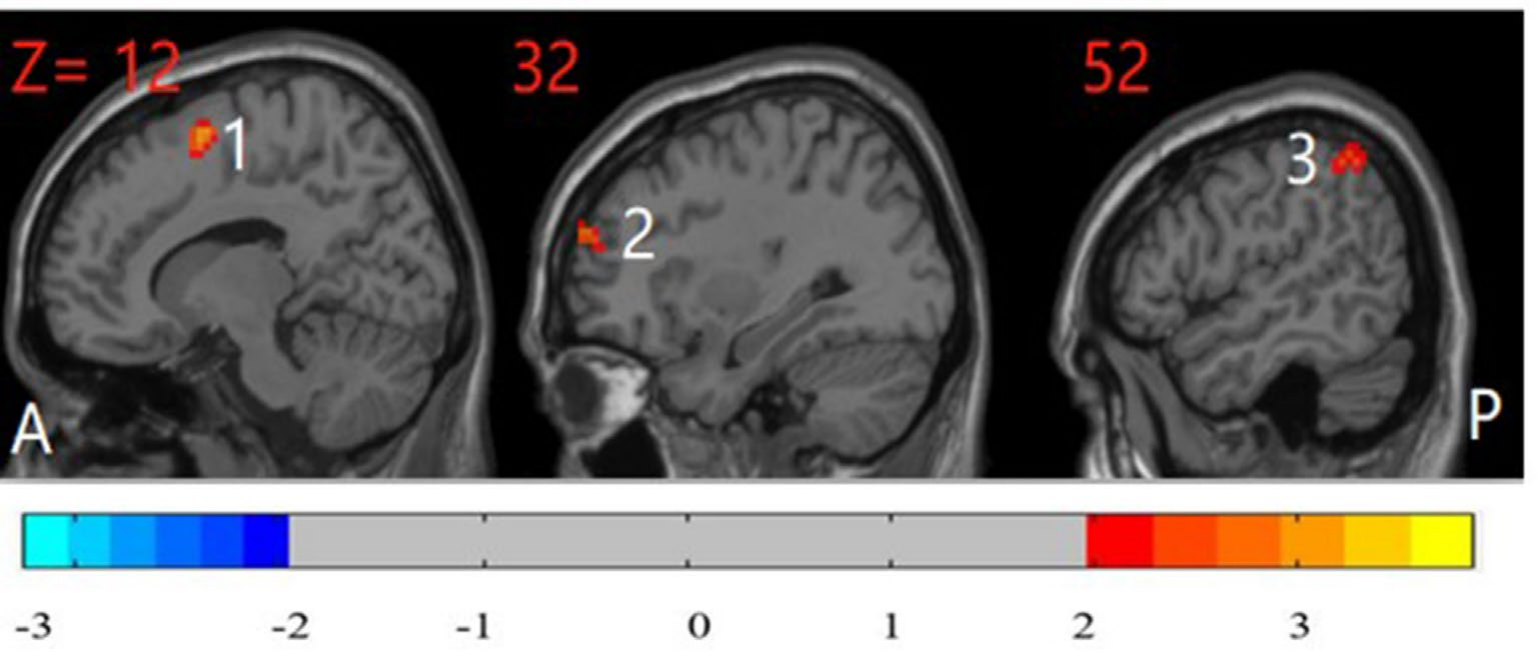
*Post hoc* test maps of ReHo between unaffected siblings of patients with OCD and healthy controls (*P* < 0.05). The unaffected siblings of OCD patients showed significantly higher ReHo in the right inferior parietal cortex, right middle frontal gyrus, and right supplementary motor area. The image shows the sagittal plane of the brain. *T*-score bars are shown on the bottom. Red and blue denote higher and lower ReHo comparing unaffected siblings of patients with healthy controls, respectively. A: anterior; P: posterior; 1: right supplementary motor area; 2: right dorsolateral prefrontal cortex; 3: right inferior parietal cortex.

The unaffected siblings of participants with OCD had significantly higher ReHo in the right inferior parietal cortex, right DLPFC, and right supplementary motor area than did HCs (*P* < 0.05, corrected) (**Figure 3**).

**Figure 4:**
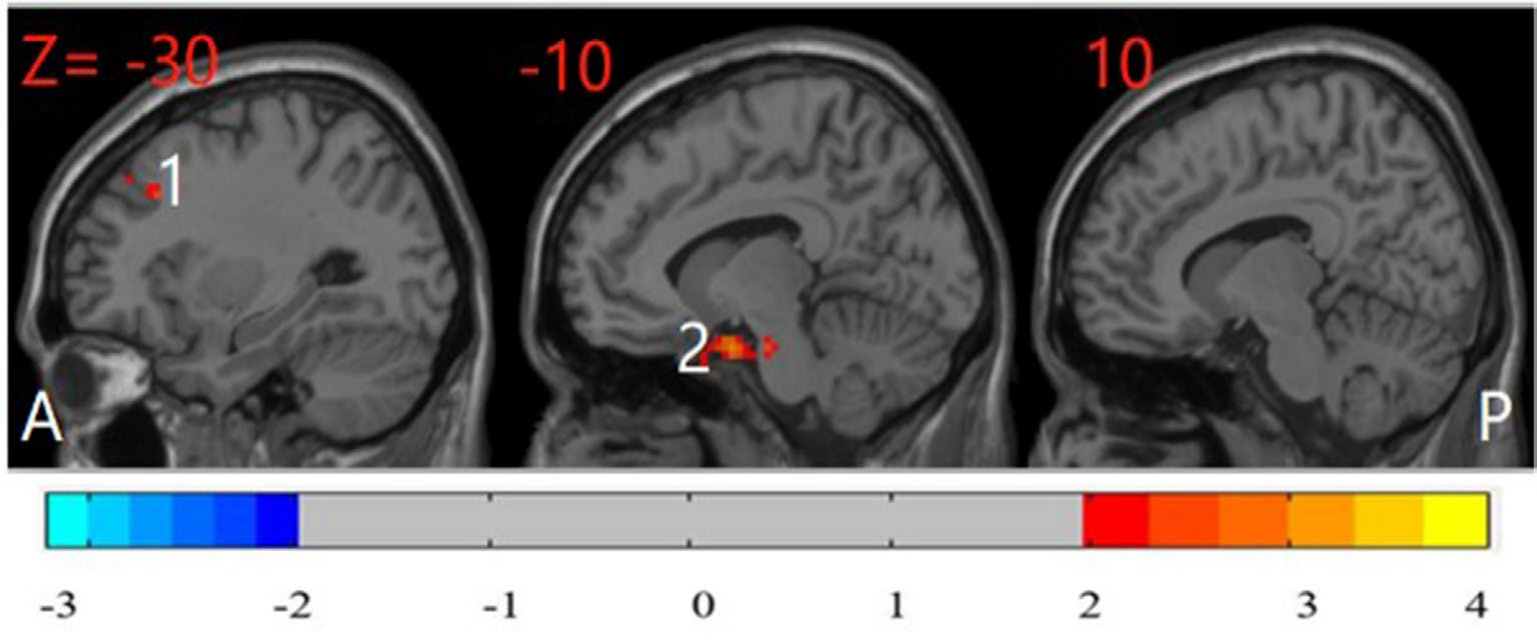
*Post hoc* test map of ReHo between patients with OCD and their unaffected siblings (*P* < 0.05). The OCD patients showed significantly higher ReHo in the left parahippocampal gyrus and left middle frontal gyrus. The image shows the sagittal plane of the brain. Red and blue denote higher and lower ReHo comparing OCD patients with their siblings, respectively. A: anterior; P: posterior; 1: left middle frontal gyrus; 2: left parahippocampal gyrus.

Compared with their unaffected siblings, participants with OCD showed significantly higher ReHo in the left PHG and left MFG (*P* < 0.05, corrected) (**Figure 4**).

On the basis of the above results, we further extracted the mean ReHo value of all regions with significant group difference between HCs, OCD patients, and their unaffected siblings to draw scatterplots (**Supplemental Figures**).

### Correlation Between Clinical Measures and ReHo in Participants With OCD

We analyzed the association of clinical symptoms (Y-BOCS scores, HAMD-17 scores, and HAM-A scores) and abnormal ReHo value in the participants with OCD; however, we did not find any correlation between their symptoms and abnormal ReHo.

## Discussion

The current study is the first to investigate brain regional homogeneity in OCD patients and their unaffected siblings. We found overlapping higher ReHo values in the right DLPFC in OCD patients and their unaffected siblings relative to HCs. This result suggests that the higher ReHo in the right DLPFC may be a potential hereditary risk indicator of OCD. Our findings provide new insights into the understanding of local synchronization in the pathology of OCD.

### Different ReHo Between OCD Patients and HCs

As mentioned above, aberrant ReHo has been detected in the prefrontal, temporal, parietal, and occipital lobes and limbic system in patients with OCD compared with HCs. In this study, patients with OCD, compared with HCs, showed significantly higher ReHo in the right DLPFC, left MFG, and right superior parietal cortex and lower ReHo in the right PHG, left inferior parietal cortex, left thalamus, and right inferior temporal cortex. These findings are consistent with previous studies and further support the revised model of OCD proposed in recent studies. Researchers recognize that these broader brain regions, including the orbito-fronto-striatal regions, limbic structures, dorsolateral frontal, parietal region, and posterior region, are involved in the pathology of OCD (31–33).

Consistent with our previous study and other studies, a correlation between severity of obsessive-compulsive symptoms and ReHo value was not found in this study (25, 30, 34). This is possibly due to the small sample size of the current study and may suggest that ReHo values do not vary with the severity of obsessive-compulsive symptoms.

### Different ReHo in Unaffected Siblings of Patients With OCD and HCs

We found higher ReHo in the right inferior parietal cortex, right DLPFC, and right supplementary motor area in unaffected siblings relative to HCs. These higher ReHo values indicate enhanced coordination of these regions. Increased functional connectivity and microstructural abnormalities within the frontoparietal region have been shown to be involved in the pathophysiology of OCD (35, 36). de Vries and colleagues using working memory task-related fMRI measured brain activity of OCD patients and their unaffected relatives. They found that frontoparietal dysfunction may constitute a neurocognitive endophenotype for OCD, while hyperconnectivity in premotor/pre-supplementary motor area is protective in unaffected first-degree relatives (14). Another task fMRI using a stop-signal task found that presupplementary motor area activity has a negative association with stop-signal reaction time in OCD patients and their siblings. They suggest that presupplementary motor area hyperactivity is a neurocognitive endophenotype of OCD (13). Norman and colleagues conducted a meta-analysis of the fMRI studies, which compared the error and inhibitory control of OCD patients and HCs, and they found hyperactivation in the bilateral premotor cortex of OCD patients relative to HCs (37). These findings suggest that the increased activity in the supplementary area may play a compensatory role in OCD and a protective role in the unaffected siblings of OCD. Hao and colleagues performed a voxel-wise meta-analysis of ReHo abnormalities in patients with OCD relative to HCs. They observed significant heterogeneity with ReHo alterations in the right supplementary motor area (19). The inconsistent results may be due to the different task design. More family designed r-s fMRI studies are needed to explore the risk factor and protective factor for OCD in the future.

### Regions With Similar and Different ReHo Between OCD Patients and Their Unaffected Siblings

Notably, compared with HCs, patients with OCD and their unaffected siblings showed overlapping increased ReHo in the right DLPFC; the patients did not differ significantly from their siblings in this region. This result suggests that the higher ReHo in the right DLPFC might be an endophenotype for OCD. The DLPFC is functionally and structurally associated with OCD symptoms (25, 32). Voxel-based morphology studies observed increased white matter and gray matter value in the MFG, including the DLPFC, of the OCD patients (38, 39). As a part of the cognitive control network, the DLPFC is involved in conflict/error monitoring in OCD patients (40). Wang and colleagues detected a relationship between ReHo and behavioral conflict adaptation in normal population, and they found that DLPFC is associated with the conflict adaptation process (41). Our previous r-s fMRI study also found higher ReHo in the right DLPFC (30). Our current findings provide further evidence for the importance of the right DLPFC in the pathophysiology of OCD. Previous studies using task-fMRI demonstrate that the abnormal activity of the orbitofrontal cortex, presupplementary motor area, and frontoparietal regions may serve as endophenotypes for OCD (12–14). One r-s fMRI study found increased functional connectivity in the left orbitofrontal cortex, caudate, and left middle temporal gyrus in both OCD patients and their first-degree relatives (17). These inconsistent findings may be partly due to different fMRI designs, including differences between the task-related state and the resting state and partly due to the different participants, including siblings or first-degree relatives of patients with OCD.

OCD patients showed increased ReHo in the left MFG relative to their unaffected siblings. Furthermore, ReHo in the left MFG of participants with OCD was also higher than that in the HCs; the siblings did not differ significantly from the HCs in this region. This result indicates that the higher ReHo in the left MFG may have a stronger association with the development of OCD. Since a previous study found reduction of gray matter volume in the left MFG of OCD patients (42), we speculate that increased ReHo in the left MFG found in our study may be the result of compensation in this region.

We found abnormal ReHo in both the left and right PHG in participants with OCD. Higher ReHo in the left PHG was found in OCD patients compared with their unaffected siblings, while lower ReHo was found in the right PHG in OCD patients compared with HCs. The PHG is the gray matter cortical region of the brain that surrounds the hippocampus and is part of the limbic system. Studies using voxel-based morphometry found increased gray matter in the PHG of OCD patients (39, 43). Task-related fMRI studies have reported that the PHG is associated with effortful memory search and retrieval, the processing of unpleasant emotions, and decision making in OCD (44, 45). Fujii and colleagues demonstrated that the activation of the bilateral PHG regions might be associated with different types of retrieval strategy in memory processes. The increased regional cerebral blood flow in the left PHG was related to a nonmatching strategy, while the increased regional cerebral blood flow in the right PHG area was related to matching (46). More research is needed to further explore the role of the left and right PHG in the pathophysiology of OCD.

### Study Limitation

We have a relatively small sample, with only 15 OCD patients and 15 unaffected siblings. The genders of the OCD patients and their siblings are not fully matched. Nevertheless, we have increased statistical power by including 30 HCs and matched their genders with those of patients and their healthy siblings. The genetic mechanisms of OCD are complex and include the interactions between genetic background, environmental factors, lifestyle, and other factors. Our current preliminary study only examined the brain regional homogeneity of participants, which has certain limitations. Hence, our results must be interpreted with caution. In the future, more studies combining neuroimaging, gene detection, and cognitive function will be more helpful for exploring the endophenotype of OCD.

## Conclusions

Our results show that the unaffected siblings of patients with OCD had significantly higher ReHo in the right inferior parietal cortex, right DLPFC, and right supplementary motor area compared to HCs. Furthermore, OCD patients and their siblings shared higher ReHo in the right DLPFC relative to HCs. This suggests that increased ReHo in the right DLPFC may serve as an endophenotype representing increased genetic risk of OCD. Our findings provide novel evidence to increase our understanding of the pathophysiology of OCD.

## Data Availability Statement

The datasets generated for this study are available on request to the corresponding author.

## Ethics Statement

This study was approved by the Research Ethics Committee at Beijing Anding Hospital, Capital Medical University. All participants provided written informed consent, in compliance with regulations of the institution and the guidelines of the Declaration of Helsinki.

## Author Contributions

ZL designed the study. JS designed the statistical methodology, analyzed data, and reviewed and edited manuscript. XY conducted data analysis and wrote the manuscript. JL, ZZ, XY, SY, PW, JG and RL collected the data. All authors critically reviewed and proofed the manuscript.

## Funding

This work was supported by a Beijing Natural Science Foundation Grant (7122082), National Natural Science Foundation of China Grant (81271493), and Beijing Municipal Administration of Hospitals Young Talents Training Program (QML20181903).

## Conflict of Interest Statement

The authors declare that the research was conducted in the absence of any commercial or financial relationships that could be construed as a potential conflict of interest.
