# Supplementary material for: Abnormal Regional Homogeneity in Patients With Obsessive-Compulsive Disorder and Their Unaffected Siblings: A Resting-State fMRI Study
Source: Front Psychiatry. 2019 Jun 28;10:452. doi: 10.3389/fpsyt.2019.00452 (PMC6609574; doi:10.3389/fpsyt.2019.00452)
Supplement: Supplementary file 1 [file Image_1.pdf]

Supplementary Figure 1-10. Scatterplots of mean ReHo values of all regions with significant group difference among OCD patients, unaffected siblings of OCD patients and HCs

Individual circles represent the mean ReHo value for each subject in each region.

OCD: obsessive - compulsive disorder; HCs: healthy controls; ReHo: regional homogeneity.

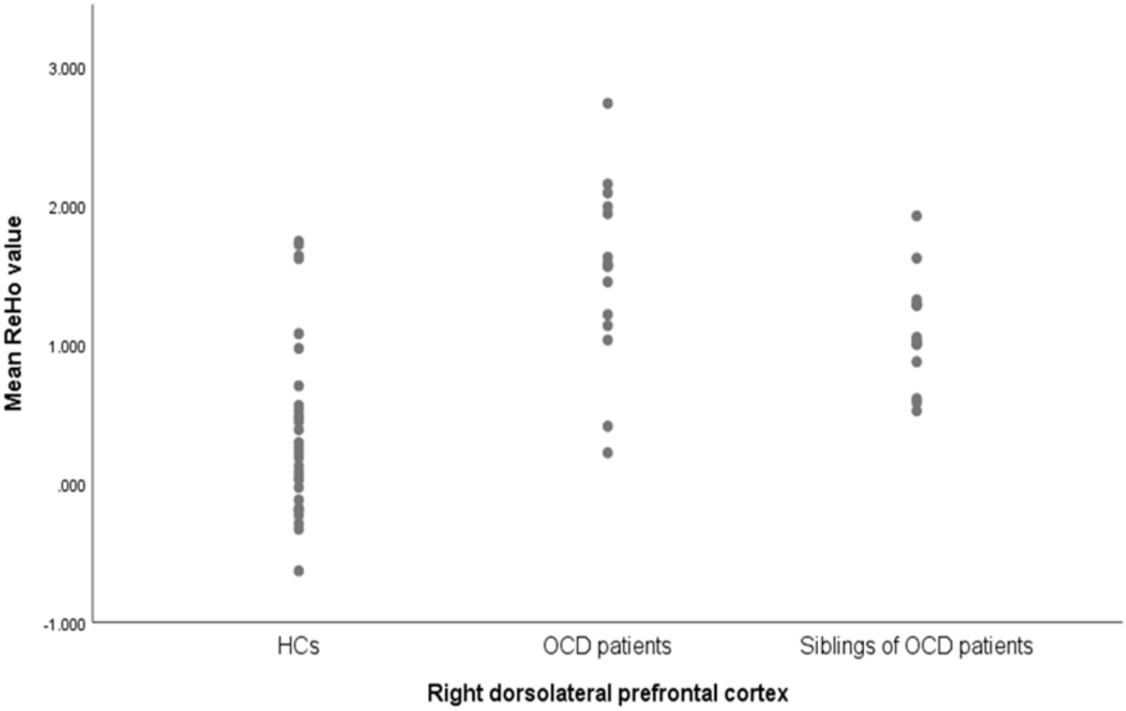

Supplement Figure.1 Scattergram of mean ReHo value in the right dorsolateral prefrontal cortex among three groups

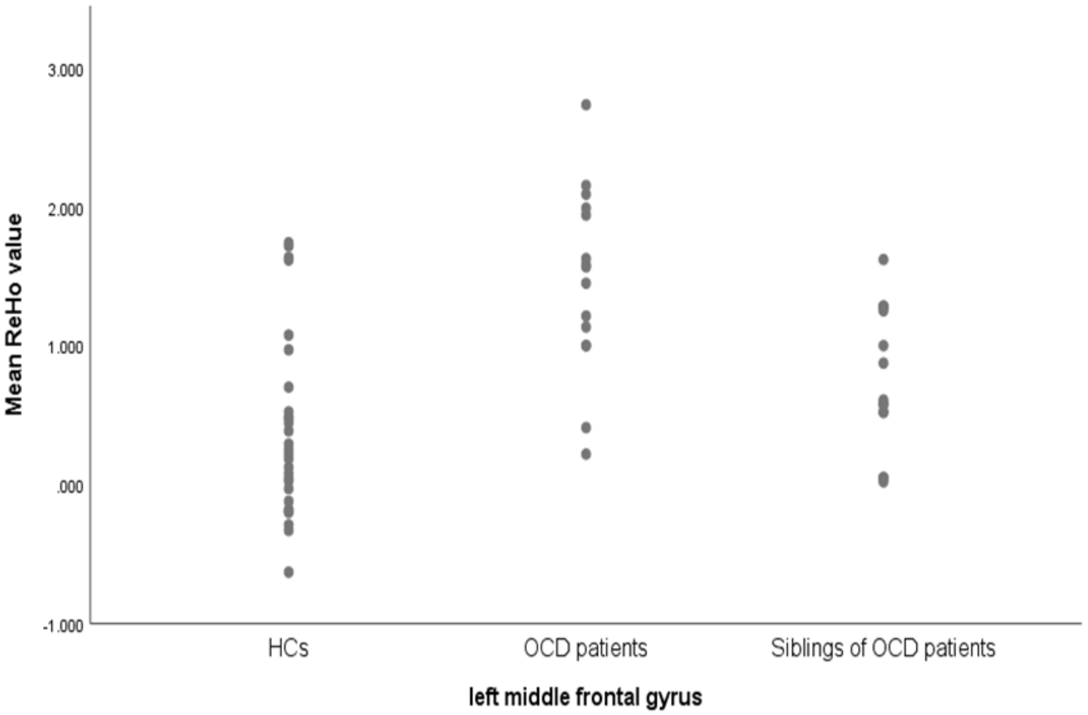

Supplement Figure.2 Scattergram of mean ReHo value in the left middle frontal gyrus among three groups

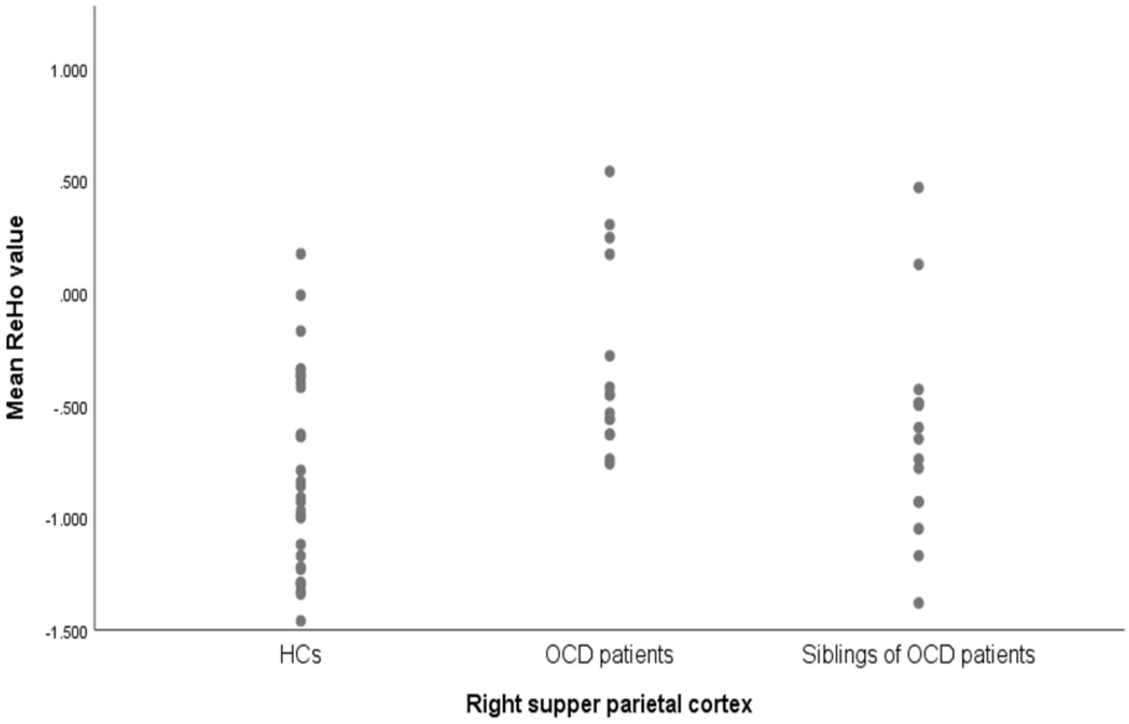

Supplement Figure.3 Scattergram of mean ReHo value in the right supper parietal cortex among three groups

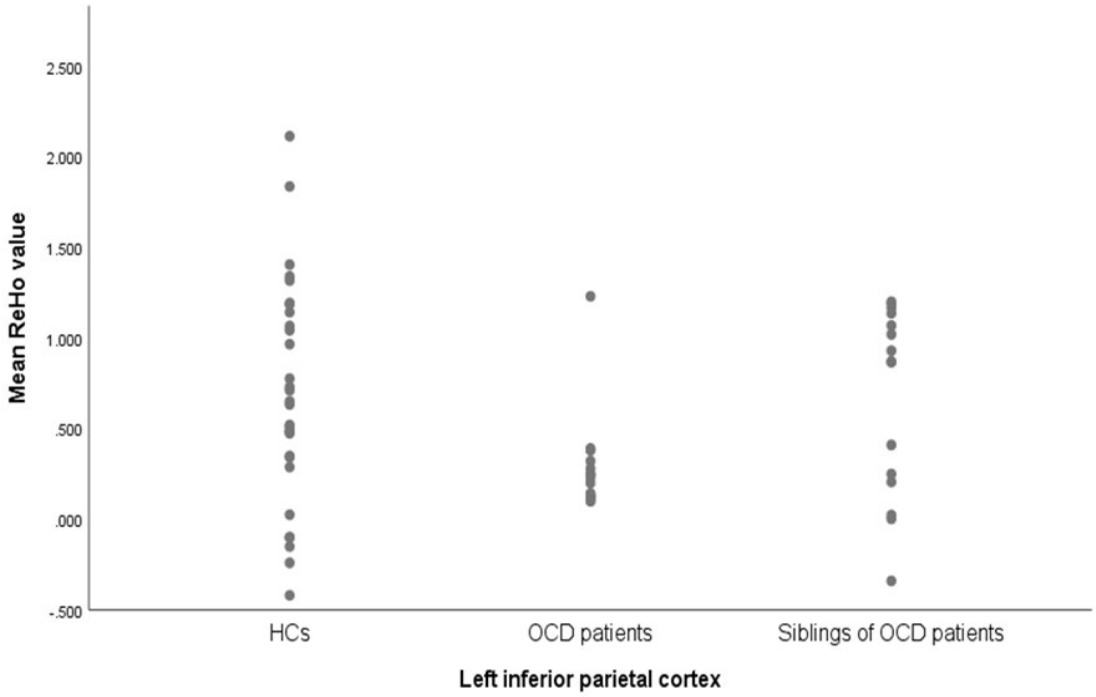

Supplement Figure.4 Scattergram of mean ReHo value in the left inferior parietal cortex among three groups

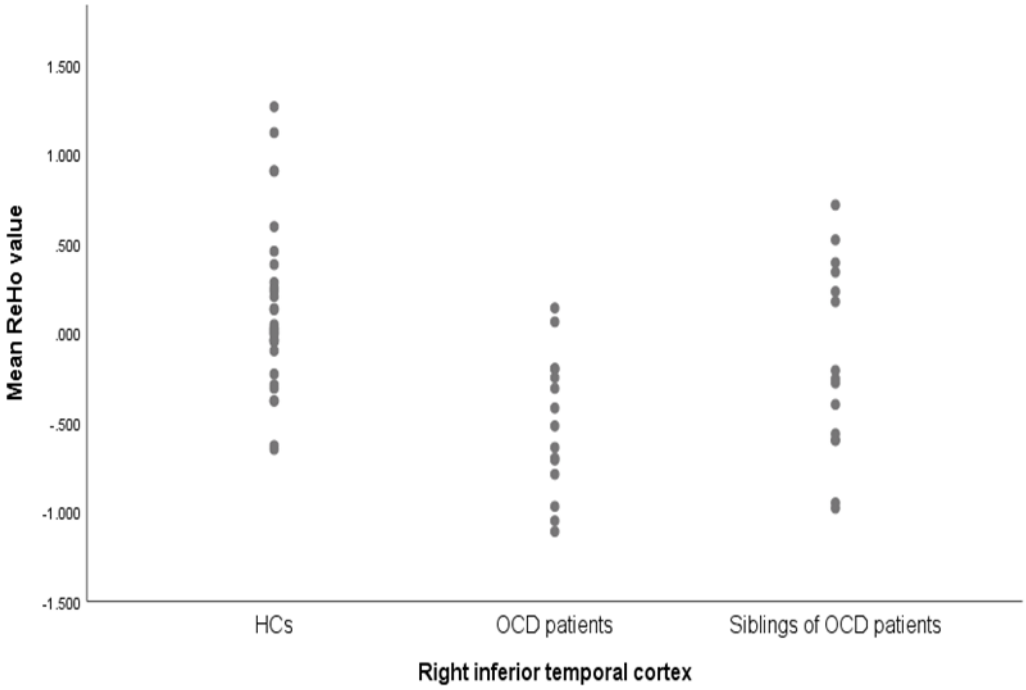

Supplementary Figure.5 Scattergram of mean ReHo values in the right inferior temporal cortex among three groups

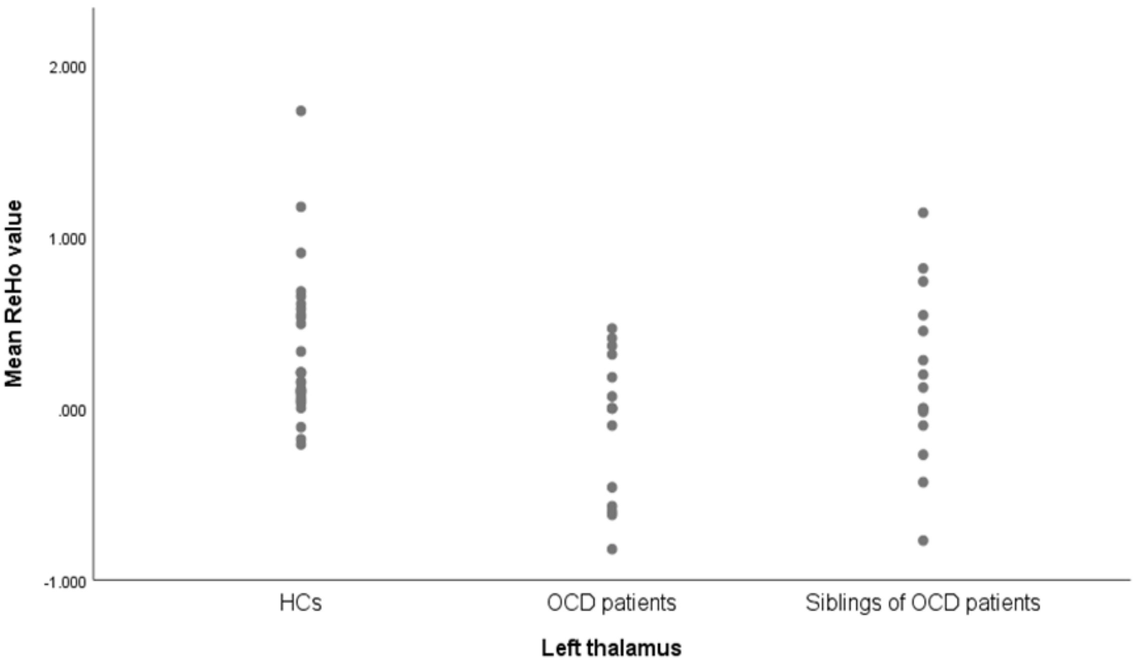

Supplement Figure.6 Scattergram of mean ReHo value in the left thalamus among three groups

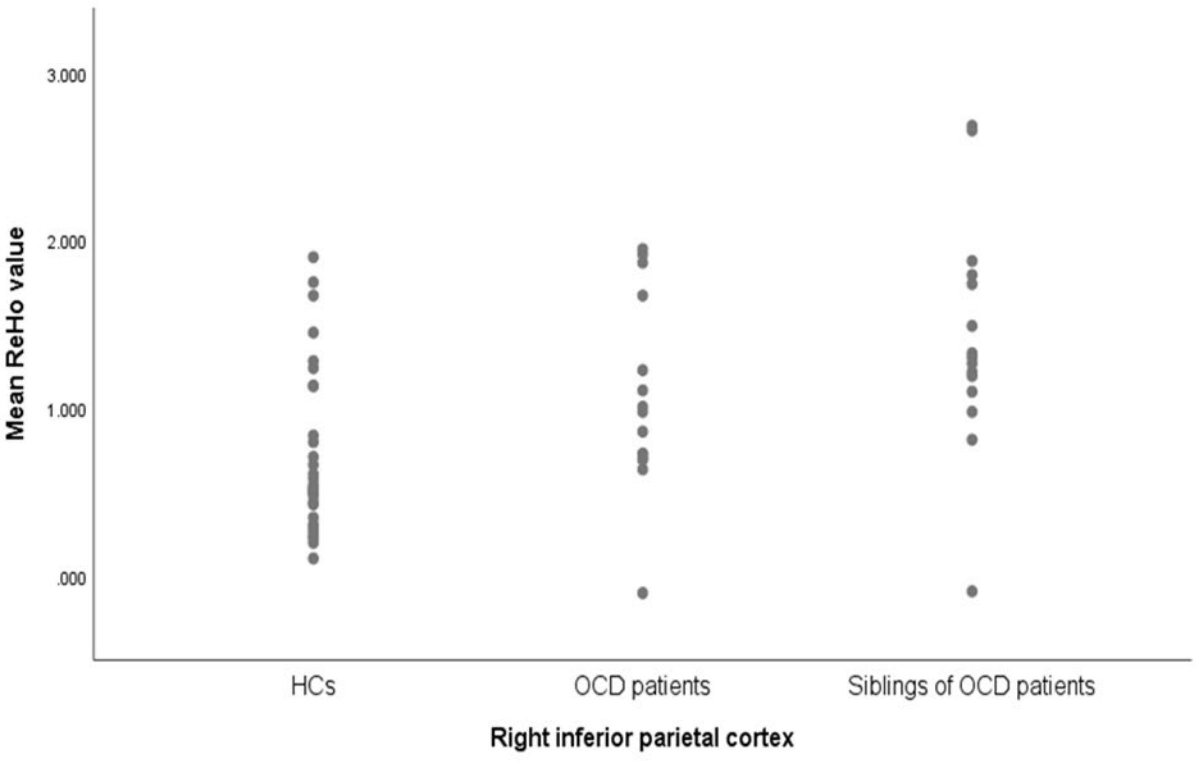

Supplement Figure.7 Scattergram of mean ReHo value in the right inferior parietal cortex among three groups

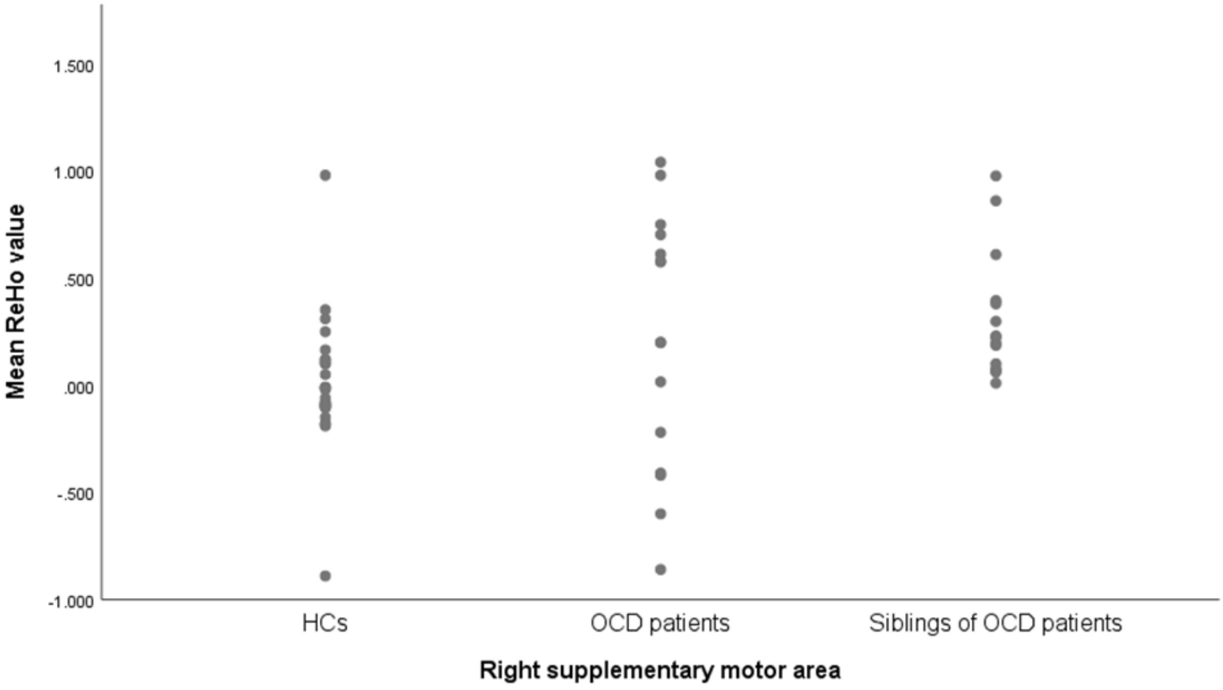

Supplement Figure.8 Scattergram of mean ReHo value in the right supplementary motor area among three groups

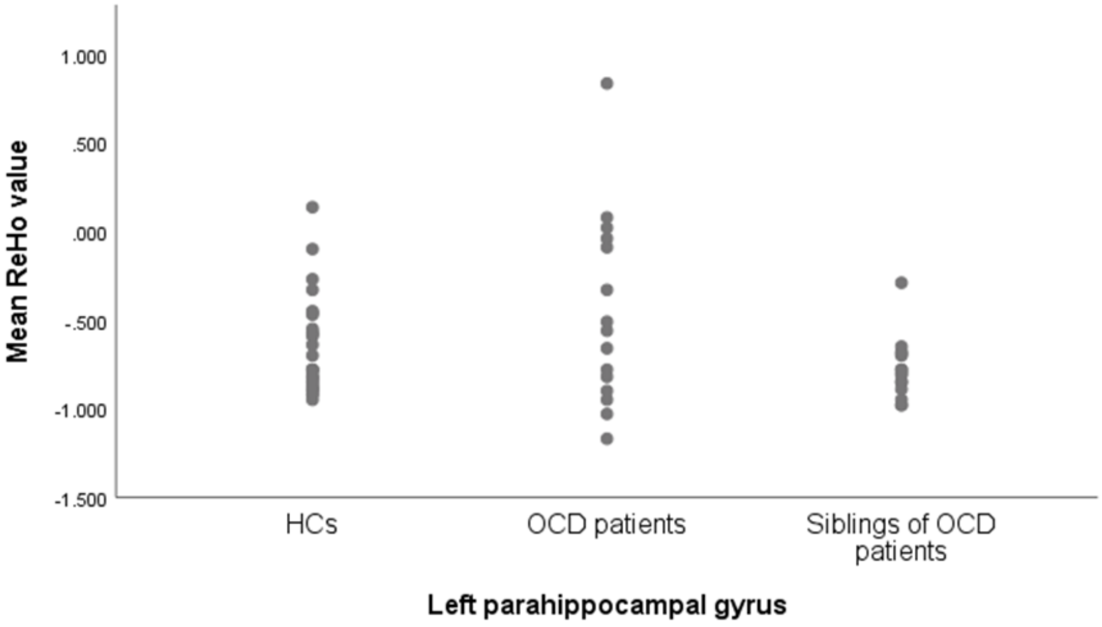

Supplement Figure.9 Scattergram of mean ReHo value in the left parahippocampal gyrus among three groups

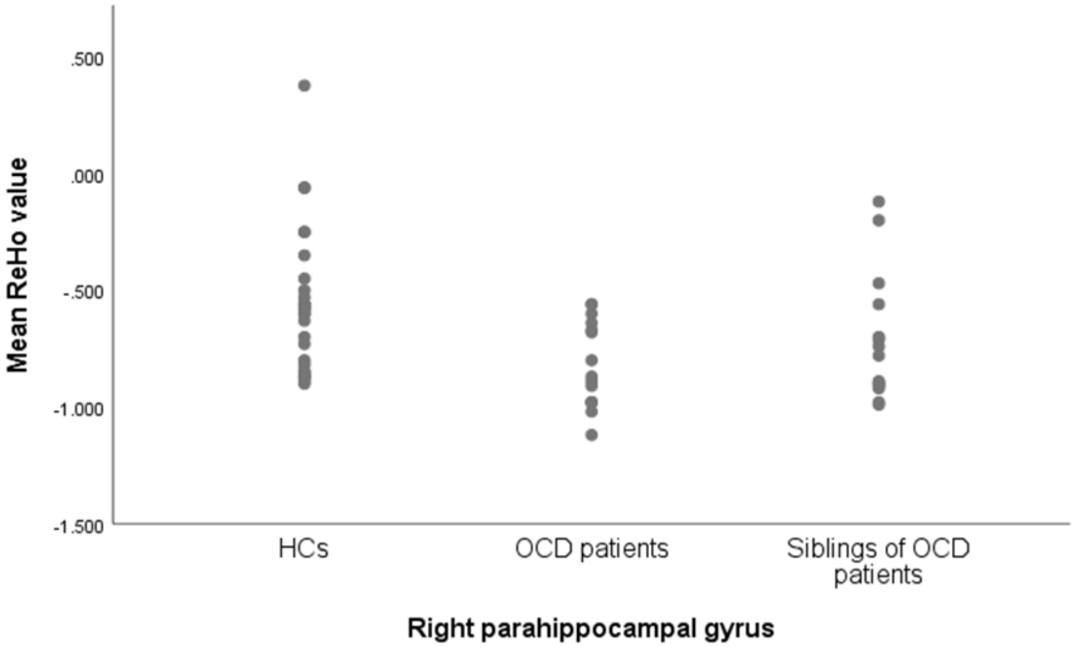

Supplement Figure.10 Scattergram of mean ReHo value in the right parahippocampal gyrus among three groups
